# Association between *IL-27* Gene Polymorphisms and Cancer Susceptibility in Asian Population: A Meta-Analysis

**DOI:** 10.31557/APJCP.2020.21.9.2507

**Published:** 2020-09

**Authors:** Abdolkarim Moazeni-Roodi, Mohammad Hashemi, Saeid Ghavami

**Affiliations:** 1 *Tropical and Communicable Diseases Research Centre, Iranshahr University of Medical Sciences, Iranshahr, Iran. *; 2 *Department of Clinical Biochemistry, School of Medicine, Iranshahr University of Medical Sciences, Iranshahr, Iran. *; 3 *Department of Clinical Biochemistry, School of Medicine, Zahedan University of Medical Sciences, Zahedan, Iran. *; 4 *Genetics of Non-communicable Disease Research Center, Zahedan University of Medical Sciences, Zahedan, Iran. *; 5 *Department of Human Anatomy and Cell Science, Rady Faculty of Health Sciences, Max Rady College of Medicine, University of Manitoba, Winnipeg, MB, Canada. *; 6 *Research Institute in Oncology and Hematology, Cancer Care Manitoba, University of Manitoba, Winnipeg, Canada. *

**Keywords:** IL-27, polymorphism, cancer, meta-analysis

## Abstract

**Background:**

Interleukin 27 (IL-27) has potent antitumor activity. Several epidemiological studies have designated that genetic variants of the IL-27 gene may contribute to various cancer susceptibility, but the data were inconclusive.

**Objective:**

The current meta-analysis aimed to address the association between* IL-27 rs153109*, *rs17855750*, and *rs181206* polymorphisms and the risk of cancer.

**Data Sources:**

Our team has selected eligible studies up to May 1, 2020, from several electronic databases, including Web of Science, PubMed, Scopus, and Google Scholar databases.

**Results:**

Our meta-analysis revealed that the carriers *rs153109 A>G* polymorphism in the *IL-27 *gene have higher risks of diseases in the heterozygous (OR=1.26, 95%CI=1.06-1.49, P=0.007, AG vs AA), homozygous (OR=1.18, 95%CI=1.01-1.37, p=0.33, GG vs AA), dominant (OR=1.25, 95%CI=1.07-1.47, P=0.006, AG+GG vs AA), and allele (OR=1.15, 95%CI=1.04-1.27, P=0.008, G vs A) genetic models. Stratified analysis by cancer type indicated that this variant was significantly associated with gastrointestinal cancer, colorectal cancer and breast cancer. The findings did not support an association between *rs17855750 T>G, rs181206 T>C* polymorphisms of *IL-27* and cancer risk.

**Conclusion:**

the current study findings suggest that IL-27 rs153109 polymorphism significantly increased the risk of cancer susceptibility. Well-designed replication in a larger independent genetic association study with larger sample sizes in diverse ethnicities is required to verify the findings.

## Introduction

Cancer, a major public health concern, remains a leading cause of morbidity and mortality worldwide (Siegel et al., 2015). While the etiologies of cancer is complicated and not fully understood, growing evidences indicating that a complex interaction between genetic and environmental factors involved in cancer development (Lichtenstein et al., 2000). 

Interleukin-27 (IL-27), belonging to the IL-12 family, is a heterodimeric cytokine comprising of two subunits, 1L-27p28 and the Epstein-Barr virus-induced gene 3 protein (EBI3), and is generally secreted by activated antigen-presenting cells (Devergne et al., 1996; Liu et al., 2008). The human *Il-27* gene (IL-27P28) is located on chromosome 16 (16p11) (Pflanz et al., 2002). It is well-known that IL-27 possesses antitumor activities against a variety of tumor types (Nagai et al., 2010; Di Carlo et al., 2014; Yoshida and Hunter, 2015; Yoshimoto et al., 2015). IL-27 is a polymorphic gene and several studies examined the association between *IL-27* gene polymorphisms and risk of various cancers including, non-small-cell lung cancer (NSCLC) (Ge and Xiao, 2016), acute lymphoblastic leukemia (ALL) (Ghavami et al., 2018), nasopharyngeal carcinoma (NPC) (Wei et al., 2009; Pan et al., 2012), colorectal cancer (CRC) (Guo et al., 2012; Huang et al., 2012; Lyu et al., 2015), prostate cancer (PCa) (Munretnam et al., 2014), papillary thyroid carcinoma (PTC) (Zhang et al., 2015; Nie et al., 2017), hepatocellular carcinoma (HCC) (Peng et al., 2013), renal cell carcinoma (RCC) (Pu et al., 2015), osteosarcoma (Tang et al., 2014), esophageal cancer (Tao et al., 2012), cervical cancer (Wang et al., 2016), endometrial cancer (Yu et al., 2016), ovarian cancer (Zhang et al., 2014b), breast cancer (Zhang et al., 2014a), glioma (Zhao et al., 2009), and bladder cancer (Zhou et al., 2015). However, the findings of these studies have been controversial. So we conducted the present meta-analysis of eligible published studies to further assess the association between the *IL-27* polymorphisms and cancer risk.

## Materials and Methods

*Identification of Eligible Studies*

Two authors independently carried out a systematic literature search in PubMed, Web of Knowledge, and Scopus for all related reports using the key words “*IL-27* or *IL27 *or *interleukin 27*” and “polymorphism or SNP or variation” and “cancer or tumor or carcinoma or malignancy or neoplasm”. The last search was updated on March 04, 2019. 

*Inclusion and Exclusion Criteria*

Studies were implemented in the current meta-analysis if they met all of the criteria: (1) Assessment of the relationship between IL-27 gene polymorphisms and cancer susceptibility; (2) Case–control studies; (3) Adequate data to estimate pooled ORs with a 95% CIs. The exclusion criteria were: (1) not a case–control study, reviews, case reports, meta-analysis, and comments; (2) duplicate publication; (3) studies with insufficient data.

*Data extraction*

The data extraction from the eligible studies was achieved independently by two researchers according to the inclusion and exclusion criteria mentioned above. In each study, the following items were collected from each study: first author’s name, publication year, country, ethnicity, cancer type, source of controls, total number of cases and controls, genotype distributions of cases and controls, and Hardy-Weinberg equilibrium (HWE), respectively.

*Statistical analysis*

All statistical analyses were achieved using Stata, version 14.1 (Stata Corporation, College Station, TX, USA). 

The HWE was evaluated for each study by the chi-square test in the control group. Pooled ORs and corresponding 95%CIs were calculated to estimate the strength of association between IL-27 gene polymorphism and cancer risk. The significance of the pooled OR was determined by Z test, in which p-value less than 0.05 was considered statistically significant.

The Q statistic test was used to check the heterogeneity among studies included in the meta-analysis. A p>0.10 indicated a lack of heterogeneity among studies, consequently the fixed effect model was used to calculate pooled OR. Otherwise, a random effects model was utilized.

Publication bias was evaluated by Begg’s funnel plot qualitatively, and Begg’s and Egger’s tests quantitatively. P-value less than 0.05 considered significant publication bias. 

Sensitivity analysis was done by removing each study in turn to measure the results stability.

**Table 1:** Characteristics of All Studies Included in the Meta-Analysis for IL-27 rs153109 Polymorphism

Author	Year	Country	Ethnicity	Cancer type	Source of control	Genotyping Method	Case/ control	Cases					Controls					HWE
rs153109 (-964 A>G)								AA	AG	GG	A	G	AA	AG	GG	A	G	
Fathi Maroufi	2018	Iran	Asian	Breast cancer	HB	PCR-RFLP	140/140	53	67	20	173	107	59	66	15	184	96	0.585
Ge	2016	China	Asian	NSCLC	HB	PCR-RFLP	388/390	115	219	54	449	327	129	213	48	471	309	0.005
Ghavami	2018	Iran	Asian	ALL	HB	PCR-RFLP	200/210	60	136	4	256	144	141	57	12	339	81	0.063
Guo	2012	China	Asian	CRC	HB	PCR-RFLP	170/160	53	84	33	190	150	75	66	19	216	104	0.449
Huang	2012	China	Asian	CRC	HB	PCR-RFLP	410/450	151	213	46	515	305	183	222	45	588	312	0.059
Lyu	2015	China	Asian	CRC	HB	PCR-RFLP	600/600	217	243	140	677	523	272	201	127	745	455	<0.001
Munretnam	2014	Malaysian	Asian	Prostate cancer	HB	Illumina’s	51/51	48		3	-	-	37		14	-	-	-
Nie	2016	China	Asian	PTC	HB	PCR-RFLP	496/629	176	252	68	604	388	279	266	84	824	434	0.107
Pan	2012	China	Asian	NPC	HB	PCR-RFLP	190/200	90	78	22	258	122	85	87	28	257	143	0.453
Peng	2013	China	Asian	HCC	HB	PCR-RFLP	107/105	38	48	21	124	90	40	46	19	126	84	0.371
Pu	2015	China	Asian	RCC	HB	PCR-RFLP	329/386	129	154	46	412	246	196	145	45	537	235	0.026
Tang	2014	China	Asian	Osteosarcoma	HB	PCR-RFLP	160/250	56	85	19	197	123	100	124	26	324	176	0.168
Tao	2012	China	Asian	ESC	HB	PCR-RFLP	426/432	163	205	58	531	321	162	219	51	543	321	0.075
Wang	2016	China	Asian	CRC	HB	PCR-RFLP	380/380	257	80	43	594	166	232	92	56	556	204	<0.001
Wei	2009	China	Asian	NPC	HB	PCR-RFLP	302/310	119	150	33	388	216	113	161	36	387	233	0.06
Yu	2016	China	Asian	Endometrial	HB	PCR-RFLP	272/320	103	132	37	338	206	161	124	35	446	194	0.139
Zhang	2014	China	Asian	Ovarian cancer	HB	PCR-RFLP	229/320	85	103	41	273	185	161	124	35	446	194	0.139
Zhang	2014	China	Asian	Breast cancer	HB	PCR-RFLP	326/460	143	156	27	442	210	185	223	52	593	327	0.213
Zhang	2015	China	Asian	PTC	HB	PCR-RFLP	664/827	287	309	68	883	445	332	399	96	1063	591	0.147
Zhao	2009	China	Asian	Glioma	HB	PCR-RFLP	210/220	79	101	30	259	161	81	112	27	274	166	0.216
Zhou	2015	China	Asian	Bladder cancer	HB	PCR-RFLP	332/499	127	160	45	414	250	229	204	66	662	336	0.058

**Figure 1:**
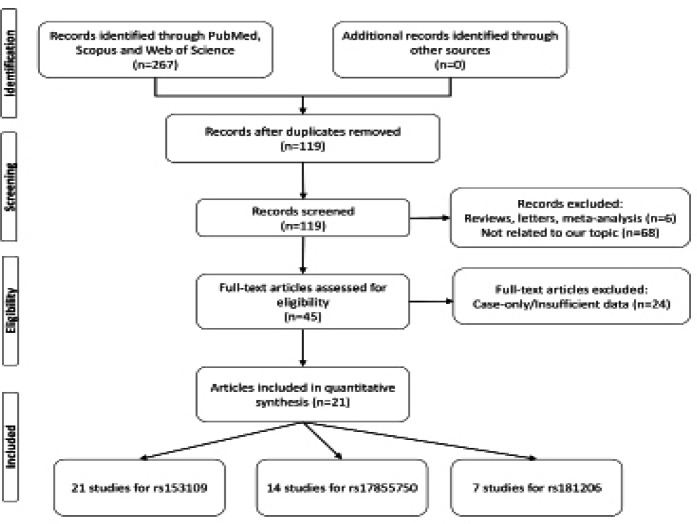
The Flow Diagram of Screening and Study Selection for Meta-Analysis

**Figure 2:**
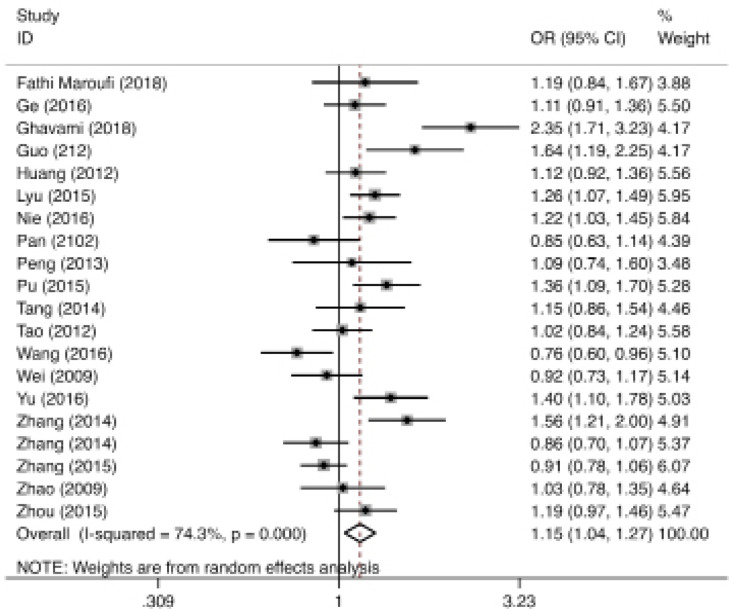
The Forest Plot for Association between rs153109 A>G Polymorphism in the IL-27 and Cancer Susceptibility for G vs A

**Table 2:** Characteristics of All Studies Included in the Meta-Analysis for IL-27 rs17855750 Polymorphism

Author	Year	Country	Ethnicity	Cancer type	Source of control	Genotyping Method	Case/	Cases					Controls					HWE
							control											
rs17855750 (2905 T>G)								TT	TG	GG	T	G	TT	TG	GG	T	G	
Ghavami	2018	Iran	Asian	ALL	HB	PCR-RFLP	200/210	34	157	9	225	175	71	124	15	266	154	<0.001
Guo	2012	China	Asian	CRC	HB	PCR-RFLP	170/160	120	41	9	281	59	122	33	5	277	43	0.151
Huang	2012	China	Asian	CRC	HB	PCR-RFLP	410/450	341	69	0	751	69	382	68	0	832	68	0.083
Nie	2016	China	Asian	PTC	HB	PCR-RFLP	496/629	382	104	10	868	124	532	96	1	1160	98	0.118
Peng	2013	China	Asian	HCC	HB	PCR-RFLP	107/105	83	21	3	187	27	72	28	5	172	38	0.304
Pu	2015	China	Asian	RCC	HB	PCR-RFLP	329/386	255	64	10	574	84	327	59	0	713	59	0.104
Tang	2014	China	Asian	Osteosarcoma	HB	PCR-RFLP	160/250	132	28	0	292	28	205	45	0	455	45	0.118
Tao	2012	China	Asian	ESC	HB	PCR-RFLP	426/432	345	81	0	771	81	355	77	0	787	77	0.042
Wang	2016	China	Asian	cervical cancer	HB	PCR-RFLP	380/380	258	76	46	592	168	182	118	80	482	278	<0.001
Wei	2009	China	Asian	NPC	HB	PCR-RFLP	302/310	247	55	0	549	55	259	51	0	569	51	0.115
Yu	2016	China	Asian	endometrial	HB	PCR-RFLP	272/320	236	33	3	505	39	267	53	0	587	53	0.106
Zhang	2014	China	Asian	Ovarian cancer	HB	PCR-RFLP	229/320	170	51	8	391	67	267	53	0	587	53	0.106
Zhao	2009	China	Asian	Glioma	HB	PCR-RFLP	210/220	169	41	0	379	41	185	35	0	405	35	0.2
Zhou	2015	China	Asian	Breast cancer	HB	PCR-RFLP	332/499	275	53	4	603	61	421	78	0	920	78	0.058

ALL, acute lymphoblastic leukemia; CRC, Colorectal cancer; ESC, esophageal cancer; PTC, papillary thyroid carcinoma; NPC, nasopharyngeal carcinoma; HCC, hepatocellular carcinoma; RCC, Renal cell carcinoma; HB, Hospital-based.

**Table 3:** Characteristics of all studies included in the meta-analysis for IL-27 rs181206 polymorphism

Author	Year	Country	Ethnicity	Cancer type	Source of control	Genotyping Method	Case/ control	Cases					Controls					HWE
rs181206 (4730 T>C)								TT	TC	CC	T	C	TT	TC	CC	T	C	
Huang	2012	China	Asian	CRC	HB	PCR-RFLP	410/450	331	79	0	741	79	373	77	0	823	77	0.047
Pan	2012	China	Asian	NPC	HB	PCR-RFLP	190/200	157	33	0	347	33	158	42	0	358	42	0.097
Tang	2014	China	Asian	Osteosarcoma	HB	PCR-RFLP	160/250	131	29	0	291	29	207	43	0	457	43	0.137
Tao	2012	China	Asian	ESC	HB	PCR-RFLP	426/432	335	91	0	761	91	354	78	0	786	78	0.039
Wang	2016	China	Asian	Cervical cancer	HB	PCR-RFLP	380/380	226	92	62	544	216	192	99	89	483	277	<0.001
Wei	2009	China	Asian	NPC	HB	PCR-RFLP	302/310	241	61	0	543	61	253	57	0	563	57	0.075
Zhao	2009	China	Asian	Glioma	HB	PCR-RFLP	210/220	166	44	0	376	44	182	38	0	402	38	0.161

CRC, Colorectal cancer; ESC, esophageal cancer; NPC, nasopharyngeal carcinoma; HB, Hospital-based

**Table 4:** The Pooled ORs and 95%CIs for the Association between IL-27 Polymorphisms and Cancer Susceptibility

Polymorphism	Genetic models	Test of association			Test of heterogeneity			Egger’s test	Begg’s test
		OR (95%CI)	Z	P	χ^2^	I^2^ (%)	P	P-value	P-value
rs153109 A>G	AG vs AA	1.26 (1.06-1.49)	2.68	0.007	94	80	<0.00001	0.364	0.436
	GG vs AA	1.18 (1.01-1.37)	2.13	0.033	33.44	43	0.021	0.835	0.679
	AG+GG vs AA	1.25 (1.07-1.47)	2.73	0.006	97.52	80	<0.00001	0.282	0.243
	GG vs AG+AA	1.05(0.93-1.19)	0.79	0.43	29.07	31	0.086	0.226	0.629
	G vs A	1.15 (1.04-1.27)	2.54	0.01	73.78	74	<0.0001	0.259	0.364
rs17855750 T>G	TG vs TT	1.11 (0.88-1.39)	0.89	0.38	53.06	75	<0.00001	0.962	0.87
	G vs T	1.13 (0.89-1.44)	1.03	0.3	81.14	84	<0.00001	0.332	0.298
rs181206 T>C	CT vs TT	1.05 (0.90-1.22)	0.62	0.53	5.73	0	0.45	0.892	0.453
	C vs T	1.00 (0.81-1.23)	0.02	0.99	14.07	57	0.03	0.125	0.453

**Table 5:** Stratified Analysis of IL-17 Polymorphisms and Cancer Susceptibility

Type of cancer	NO.	AG vs AA		GG vs AA		AG+GG vs AA		GG vs AG+AA		G vs A	
		OR (95%CI)	P	OR (95%CI)	P	OR (95%CI)	P	OR (95%CI)	P	OR (95%CI)	P
rs153109 A>G											
GI cancer	5	1.25 (0.99-1.58)	0.6	1.35 (1.11-1.65)	0.003	1.28 (1.02-1.59)	0.03	1.19 (0.99-1.43)	0.06	1.18 (1.07-1.30)	0.007
Colorectal cancer	3	1.40 (1.17-1.67)	0.0003	1.45 (1.14-1.83)	0.002	1.41 (1.19-1.66)	<0.0001	1.20 (0.97-1.49)	0.09	1.25 (1.12-1.41)	0.0001
Breast cancer	2	1.40 (1.02-1.91)	0.04	1.95 (1.27-3.01)	0.002	1.49 (1.05-2.10)	0.02	1.46 (1.10-2.46)	0.02	1.40 (1.07-1.81)	0.01
Esophageal cancer	2	1.20 (0.67-2.16)	0.55	0.81 (0.54-1.23)	0.32	0.86 (0.67-1.10)	0.23	0.88 (0.60-1.29)	0.51	0.90 (0.75-1.08)	0.24
rs17855750 T>G		TG vs TT		GG vs TT		TG+GG vs TT		GG vs TG+TT		G vs T	
GI cancer	4	1.07 (0.86-1.32)	0.54	_	_	_	_	_	_	1.06 (0.83-1.35)	0.65
Colorectal cancer	2	1.18 (0.87-1.59)	0.29	_	_	_	_	_	_	1.21 (0.92-1.59)	0.17
rs181206 T>C		CT vs TT		CC VS TT		CT+CC VS TT		CC VS CT+TT		C VS T	
Nasopharyngeal carcinoma	2	0.98 (0.70-1.37)	0.89	_	_	_	_	_	_	0.98 (0.73-1.33)	0.91

**Figure 3:**
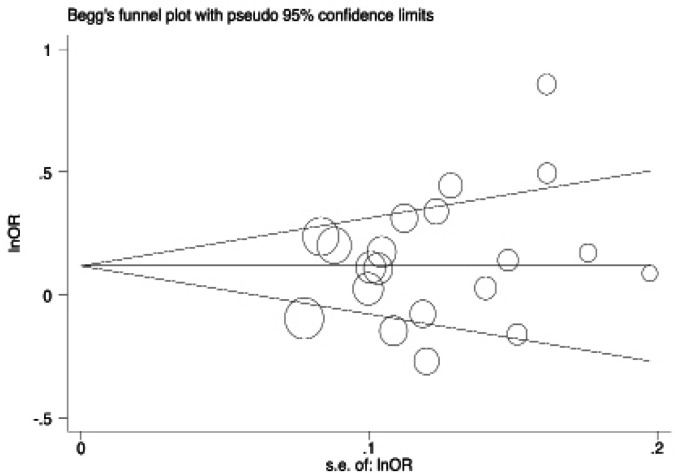
Begg’s Funnel Plot for Publication Bias Test for IL-27 rs153109 A>G Polymorphism and Cancer Risk for G vs A

**Figure 4:**
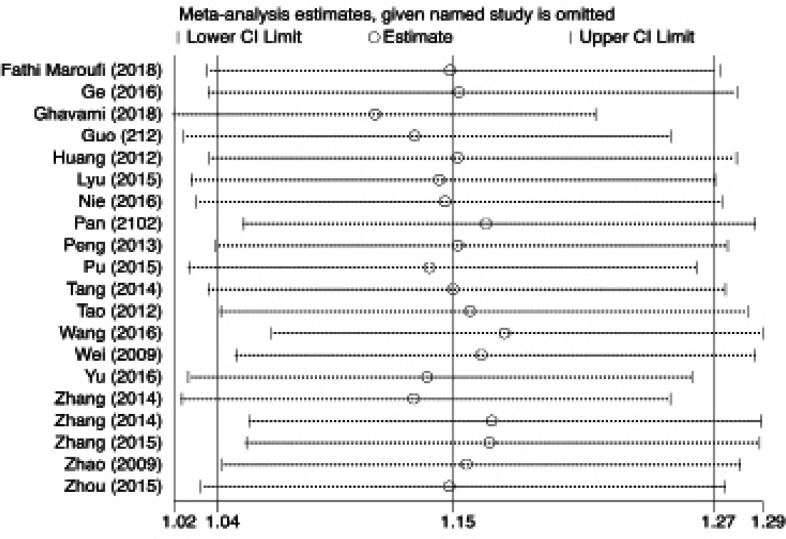
Sensitivity Analyses for Association between IL-27 rs153109 A>G Polymorphism and Cancer Risk for G vs A

## Results

*Study Characteristics*

In the current study, according to the inclusion and exclusion criteria, ultimately 20 case-control studies included in the meta-analysis. For *rs153109*, 21 studies containing 6,331 cases and 7,287 controls for were included in the quantitative analysis. Regarding *rs17855750* variant, 4,023 cases and 4,671 controls from 14 studies and for *rs181206* polymorphism, 2,078 cases and 2,242 controls from were 7 studies were included in the meta-analysis. The characteristics of the included studies are summarized in Table 1, Table 2 and Table 3. 

*Association between IL-27 polymorphisms and cancer risk*

The frequency distribution of genotype and allele of the *IL-27 *polymorphisms in cases and controls are indicated in Table 1, Table 2 and Table 3. Table 4 shows the main findings of our meta-analysis. Regarding *rs153109 A>G *variant, 21 independent studies were pooled and a random effect was applied due to the presence of significant heterogeneity. The finding revealed that* rs153109* variant significantly increased the risk of cancer in heterozygous (OR=1.26, 95%CI=1.06-1.49, P=0.007, AG vs AA), homozygous (OR=1.18, 95%CI=1.01-1.37, p=0.33, GG vs AA), dominant (OR=1.25, 95%CI=1.07-1.47, P=0.006, AG+GG vs AA), and allele (OR=1.15, 95%CI=1.04-1.27, P=0.008, G vs A) genetic models (Figure 1 and Table 4). 

Stratified analysis by cancer type (Table 5) revealed that rs153109 significantly increased the risk of gastrointestinal (GI) cancer in homozygous (OR=1.35, 95%CI=1.11-1.65, p=0.003), dominant (OR=1.28, 95%CI=1.02-1.59, p=0.030) and allele (OR=1.18, 95%CI=1.07-1.30, p=0.007) genetic models. Besides, the variant was significantly associated with colorectal cancer (CRC) and breast cancer susceptibility in all genetic model tested (Table 5).

The findings showed that rs17855750 T>G, and rs181206 T>C variants were not associated with cancer risk (Table 4).

*Heterogeneity and publication bias*

Heterogeneity among studies involved in the meta-analysis is presented in Table 4. The findings indicated that heterogeneity exist among studies and random-effects was used to estimate the pooled OR and 95% CI (Figure 2 and Table 4). 

Begg’s funnel plot, Begg’s test, and Egger’s test (Figure 3, and Table 4) indicated no evidence of significant publication bias. 

*Sensitivity analysis*

After doing the sensitivity analyses, the pooled ORs showed no statistically significant changes in heterozygous, dominant, recessive, and allele representing that our findings are stable and reliable in overall analysis (Figure 4). 

## Discussion

Several studies examined the association between IL-27 polymorphisms and the risk of various cancer (Wei et al., 2009; Zhao et al., 2009; Guo et al., 2012; Huang et al., 2012; Pan et al., 2012; Tao et al., 2012; Peng et al., 2013; Munretnam et al., 2014; Tang et al., 2014; Zhang et al., 2014a; Zhang et al., 2014b; Lyu et al., 2015; Pu et al., 2015; Zhang et al., 2015; Zhou et al., 2015; Ge and Xiao, 2016; Wang et al., 2016; Yu et al., 2016; Nie et al., 2017; Ghavami et al., 2018). The findings were controversial, though it is difficult to clarify the inconsistent findings. In this study, we conducted a comprehensive meta-analysis of all eligible studies to derive a more precise estimation of the relationship between *IL-27* polymorphism and cancer risk. After pooling all the available data, the finding suggested that* IL-27 rs153109* (-964 A>G) significantly increased the risk of overall cancer. Stratified analysis by cancer type designated that *rs153109* polymorphism was positively associated with GI cancer, CRC and BC susceptibility. The findings did not support an association between *rs17855750* (2905 T>G) and *rs181206* (4730 T>C) polymorphisms and cancer susceptibility. 

The molecular mechanisms by which variant increased the risk of cancer have not been clarified. It seems reasonable to speculate that *rs153109* polymorphism effects on *IL-27* expression. There is increasing evidence that the expression level of *IL-27p28 *gene is decreased in various cancers including epithelial ovarian cancer (Zhang et al., 2014b), bladder cancer (Zhou et al., 2015), esophageal cancer (Tao et al., 2012), osteosarcoma (Tang et al., 2014), as well as papillary thyroid cancer (Zhang et al., 2015). It has been shown that *IL-27* differentially regulates the expression of several microRNAs (miR) such as *hsa-miR-7702, hsa-miR-7704, hsa-miR-7704 hsa-miR-6852*, and *hsa-miR-6852* (Swaminathan et al., 2013; Poudyal et al., 2018).

Cytokines, secreted by cells of innate and adaptive immune systems, are small proteins that play key roles in immune responses. *IL-27* is produced early after activation by antigen-presenting cells, including monocyte-derived dendritic cells and lipopolysaccharide-stimulated monocytes (Chiyo et al., 2004; Owaki et al., 2005). *IL-27 *mediates its biological functions via a heterodimeric receptor consisting of WSX-1 and glycoprotein 130 (gp130) (Pflanz et al., 2004). Binding of *IL-27* to its receptor activates Janus kinase (JAK)-signal transducer and activator of transcription (STAT) and mitogen-activated protein kinase (MAPK) signaling (Kastelein et al., 2007). *IL-27* has potent antitumor activity (Hisada et al., 2004; Chiyo et al., 2005). It exerts antitumor activity by promoting the generation of myeloid progenitor cells that can differentiate into M1 macrophages (Chiba et al., 2018). In addition,* IL-27* synergizes with *IL-12* to potentiate IFN-γ production by activated naive T-cell and natural killer-cell populations (Pflanz et al., 2002). Beside, *IL-27* is a major stimulus of *IL-10* production by T cell (Hunter and Kastelein, 2012; Liu et al., 2013). 

Some limitations should be addressed in our meta-analysis. First, heterogeneity among studies was observed which may be result of difference of ethnicity, source of control, and cancer type. Second, this study focused on the impact of limited variants of *IL-27* and cancer susceptibility. Gene-gene as well as gene-environment interactions could influence cancer risk. Third, all studies were from Asian populations; consequently, conclusions drawn may not apply to the all population. Finally, the sample sizes of the studies are relatively small particularly in subgroup analysis. So, the results should be interpreted with caution.

In conclusion, the findings of this meta-analysis provide evidence for an association between *IL-27 rs153109* polymorphism and cancer risk. Well-designed studies with larger sample sizes in various cancer and different ethnicities are still needed in the future.
